# Clinicopathological correlations in lupus nephritis; a single center experience

**DOI:** 10.12860/jnp.2014.22

**Published:** 2014-04-12

**Authors:** Hamid Nasri, Ali Ahmadi, Azar Baradaran, Ali Momeni, Parto Nasri, Saeed Mardani, Mahmood Rafieian-kopaei, Muhammed Mubarak

**Affiliations:** ^1^Department of Nephrology, Division of Nephropathology, Isfahan University of Medical Sciences, Isfahan, Iran; ^2^Department of Epidemiology, Shahid Beheshti University of Medical Sciences, Tehran, Iran; ^3^Department of Pathology, Isfahan University of Medical Sciences, Isfahan, Iran; ^4^Department of Nephrology, Shahrekord University of Medical Sciences, Shahrekord, Iran; ^5^Medical Plants Research Center, Shahrekord University of Medical Sciences, Sharhekord, Iran; ^6^Department of Histopathology, Sindh Institute of Urology and Transplantation (SIUT), Karachi, Pakistan

**Keywords:** Crescents, Lupus nephritis, Proteinuria, Renal biopsy

## Abstract

*Background:* Renal biopsies play an important role in the diagnosis, management and prognosis of patients with lupus nephritis (LN).

*Objectives:* To determine the correlation between the pathological features of LN and the demographic, clinical and laboratory parameters.

*Patients and Methods:* This retrospective study was conducted from 2008 to 2014 on all consecutive cases of biopsy-proven LN at a nephropathology laboratory in Iran. The demographic, clinical and laboratory data were obtained from patients^’^ files and the biopsy findings from the original biopsy request forms.

*Results:* Of the 84 patients enrolled, 69 (82.2%) were females and 15 (17.8%) males. The mean age was 32.7±12 years. The mean serum creatinine was 1.5±0.94 mg/dl and the mean 24-h proteinuria, 1.6±1.9 grams. The majority of cases belonged to classes III and IV. The extracapillary proliferation was found in 42.86% of biopsies and endocapillary proliferation in 66.67% of biopsies. Patients of class IV-LN had a higher mean serum value of creatinine in comparison to class III-LN (2.19±1.09 versus 1.2±0.56 mg/dl; *p*= 0.0001). There was no significant difference of 24-h proteinuria between the two classes (*p*= 0.882). A significant association of serum creatinine with 24-h proteinuria (*p*= 0.041) was seen. Serum creatinine had significant positive correlation with activity percent (*p*< 0.001), and chronicity percent (*p*= 0.006), and also with proportion of glomeruli with crescents (*p* < 0.001). While there was a significant relationship of proteinuria with chronicity percent (*p*= 0.001), this association for activity percent was not significant (*p*= 0.094). Furthermore, the association of proteinuria with totally sclerotic glomeruli and proportion of crescents was not statistically significant (*p*= 0.186 and *p*= 0.0145 respectively).

*Conclusions:* In conclusion, the results from our study on biopsy-proven cases of LN largely concur with the previously reported studies from Iran and other parts of the world.

## 1. Introduction

Kidney involvement is one of the most severe complications of systemic lupus erythematosus (SLE). Around 60% of adults and 80% of children with SLE develop lupus nephritis (LN) (1-3). Renal biopsies play an important role in the diagnosis, management and prognosis of patients with LN. The morphologic lesions of injury in the glomeruli of a kidney biopsy from a patient with SLE, mostly include the proliferative lesions in the mesangial, endocapillary and extracapillary areas. There is generally a good correlation between the morphological lesions and clinical and laboratory features, however, this is not perfect. Hence, there is a need for performing renal biopsies to accurately determine the extent of parenchymal damage in LN and to guide treatment. Numerous studies are available on this subject mainly from developed countries (4-11). However, there are very few publications currently available concerning the demographic, clinical, and pathological features of LN and their correlation in Iran (4,5). This paucity of knowledge in this particular area prompted us to undertake this retrospective analysis of biopsy-proven LN at a single reference nephropathology laboratory in Iran.

## 2. Objectives

In this study, we aimed to conduct a retrospective analysis of the pathological features of biopsy-proven LN patients and to correlate these with various demographic, clinical and laboratory features at the time of biopsy. We sought to conduct this investigation as per International Society of Nephrology/Renal Pathology Society (ISN/RPS) 2003 classification of LN.

## 3. Patients and Methods

### 3.1. Study and setting

The present investigation is a retrospective analysis of renal biopsies of 84 patients with LN, who were referred by either nephrologists or rheumatologists. The patients fulfilled the revised American College of Rheumatology (ACR) criteria for SLE (6) as determined by their physicians. Renal biopsy-confirmed LN cases were classified according to the 2003 ISN/RPS LN classification (4).

The study was conducted from January 2008 to January 2014. All performed renal biopsies were sent to a reference laboratory. The kidney morphologic lesions were reviewed by a single nephropathologist.

### 3.2. Definition of lupus nephritis (LN)

The definitive diagnosis of LN was based on clinical and laboratory findings and finally by light microscopy (LM) and immunofluorescence (IF) study which comprised significant positive C1q (more than 2+ intensity) accompanied by IgG, IgA, IgM and C3 deposits (full-house pattern), which was semi-quantitatively graded from 0 to 3+ according to the intensity of fluorescence (4,5).

### 3.3. Definition of morphologic lesions

Morphologic lesions by LM, were evaluated according to the 2003 ISN/RPS LN classification (4). Briefly, class I was diagnosed with minimal mesangial alterations on LM, class II with mesangial proliferation and both featuring immune deposits in the mesangium on IF microscopy. Class III of LN was diagnosed when the proliferative or inflammatory lesions involved less than 50% of all glomeruli, while, class IV LN was diagnosed in the presence of diffuse glomerulonephritis (involving ≥50% of total number of glomeruli) either with global (class IV-G) or segmental (class IV-S) involvement, and also with percent of active or chronic lesions (4). Class VI was diagnosed when ≥90% glomeruli were sclerosed without residual activity (4).

### 3.4. Demographic, clinical and laboratory data

We reviewed the patients’ medical records to obtain various demographic, clinical and laboratory data at the time of their biopsies and for follow-up. We gathered the following data at the time of biopsy: race, gender, age, serum creatinine and proteinuria (based on a 24-hour urine collection).

### 3.5. Ethical issues

(1) The research followed the tenets of the Declaration of Helsinki; (2) informed consent was obtained; and (3) the research was approved by the institutional review board.

### 3.6. Statistical analysis

All continuous values were expressed as mean ± SD and categorical variables were presented as percentage. The Student’s t-test was employed to compare differences between the means of continuous variables in two groups. Pearson’s correlation and Chi square tests were used to compare frequency variables and correlation among different variables. Data were analyzed by Stata software (Stata Corp. 2011. *Stata Statistical Software: Release 12*. College Station, TX: Stata Corp LP). P value of less than 0.05 (*p*<0.05) was assumed to be significant.

## 4. Results

In our observational study, we enrolled a total of 84 LN patients’ biopsies. Of the 84 patients, 69 (82.2%) were females and 15 (17.8%) males, with a female to male ratio of 4.6:1. The mean age of all patients was 32.7±12 years. The mean serum creatinine at the time of biopsy was 1.5±0.94 mg/dl (range: 0.7 to 5 mg/dl) and the mean 24-h urinary protein excretion was 1.6±1.9 grams (range: 180 mg to 15.7 grams). The main demographic, clinical and laboratory findings of the study cohort are shown in Table 1 .

**Table 1:** The main demographic, and laboratory features of all patients with lupus nephritis.

Total number of patients	84
Mean age±SD (in years)	32.7±12
Gender	
Males	15 (17.8%)
Females	69 (82.2%)
24-h urinary protein excretion (g)	1.6±1.9

The renal biopsies were adequate for rendering specific diagnosis of LN in all 84 cases. The mean total number of glomeruli per biopsy specimen was 16.9±9.1 (range: 2 to 48) and the mean number of globally sclerosed glomeruli was 1.5±2.8 (range: 0 to 18). Table 2  shows the distribution of renal biopsies according to ISN/RPS 2003 classification. It is apparent from this table that the majority of cases belong to class III and IV. Global glomerular involvement was seen in 39 (46.4%) cases. Most of these cases (n=37) belonged to class IV LN. Segmental glomerular involvement was seen in 22 (26.2%) cases. The extracapillary proliferative lesions (crescents) were found in 42.86% of biopsies and endocapillary proliferation in 66.67% of biopsies (Table 3).

**Table 2:** The frequency distribution of different classes of lupus nephritis according to International Society of Nephrology/Renal Pathology Society (ISN/RPS) 2003 classification.

**ISN/RPS classes**	**Frequency**	**Percent**
Class I	1	1.19
Class II	9	10.71
Class III	26	30.95
Class IV	37	44.05
Class V	10	11.9
Class VI	1	1.19

**Table 3:** The main glomerular pathological features on renal biopsies of all patients with lupus nephritis.

Total number of glomeruli, mean±SD	16.9±9.1
Globally sclerosed glomeruli, mean±SD	1.5±2.8
Segmental glomerular involvement, %	26.2%
Global glomerular involvement, %	46.4%
Crescent formation, %	42.86%
Number of glomeruli with crescents, mean±SD	1.1±2
Endocapillary proliferation, %	66.67%

**Figure 1:**
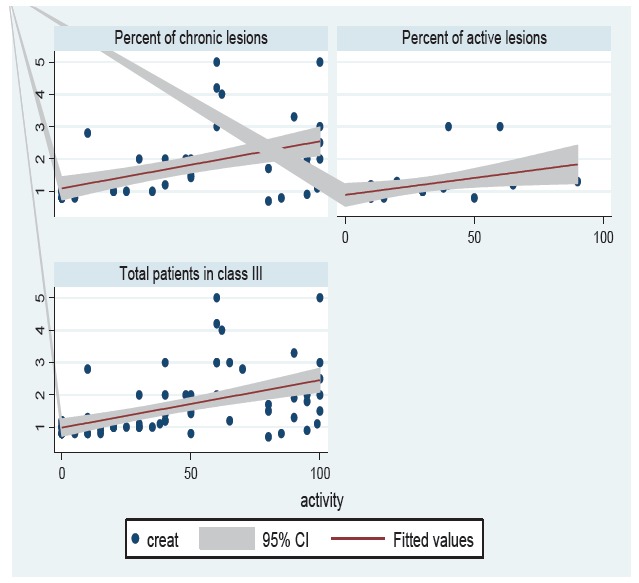
The correlation of serum creatinine with the activity and chronicity percents in patients with lupus nephritis class III.

**Figure 2:**
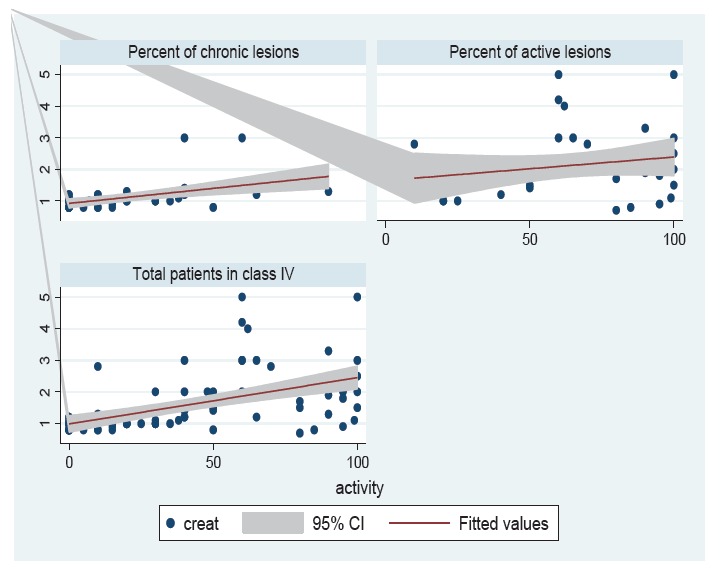
The correlation of serum creatinine with the activity and chronicity percents in patients with lupus nephritis class IV.

Regarding the activity and chronicity of glomerular lesions, in this study we found that 30.95% of patients in class III-LN had active (A) lesions, while 69.05% of lesions were chronic (C) or active/chronic (A/C). Likewise, 64.16% of patients in class IV-LN had active (A) lesions, whereas 35.84% of lesions were chronic (C) or active/chronic (A/C). Table 4  shows that 64±28 of class IV versus 30±23 of class III, had active glomerular lesions and this was statistically significant (*p*= 0.0001). Since, the most common classes of LN were class III and IV, we carried out correlation analysis for these classes of LN. There was no significant difference in the mean number of totally sclerotic glomeruli between class III and IV LN biopsies (*p*= 0.152). Patients of class IV-LN had a higher mean serum value of creatinine in comparison to class III- LN (2.19±1.09 *versus * 1.2±0.56 mg/dl; *p*= 0.0001). Additionally, there was no significant difference of 24-h urinary protein excretion between the two classes (*p*= 0.882). The mean serum creatinine was higher in male patients than in females, but was not statistically significant (1.62±1.2 versus 1.52±0.88 mg/dl; *p*= 0.709). The mean number of totally sclerosed glomeruli in male patients was more than in female patients (7.3±1.1 versus 1.65± 3; *p*= 0.001). In this study, a significant association of serum creatinine with the degree of 24-h proteinuria (*p*= 0.041) was seen. The association of serum creatinine with globally sclerotic glomeruli was not statistically significant (*p*= 0.115). Serum creatinine had significant positive correlation with activity percent (*p*< 0.001), and chronicity percent (*p*= 0.006) (Figures 1 and 2 ), and also with proportion of glomeruli with crescents (*p*< 0.001).While, there was a significant relationship of proteinuria with chronicity percent (*p*= 0.001), this association for activity percent was not significant (*p*= 0.094). Furthermore, the association of proteinuria with totally sclerotic glomeruli and proportion of crescents was not statistically significant (*p*= 0.186 and *p*= 0.0145, respectively).

**Table 4:** The main demographic, laboratory and pathology features of all patients with lupus nephritis and in patients with class III and IV according to gender of patients.

	Total patients	Class III			Class IV			*P value
		Total	Female	Male	Total	Female	Male	
Age (years)	32.7±12	30.7±15	30.3±12	32±24	32.8±12	32.7±11	33.5±15	0.545
Creatinine (mg/dl)	1.5±0.9	1.2±0.5	1.1±0.4	1.4±0.8	2.1±1	2.1±0.9	2±1.5	0.0001
Proteinuria/day (mg)	2600± 1939	2842± 2972	2338± 1571	4519± 5513	2761 ± 1238	2864 ± 1284	2317 ± 972	0.882
Mean number of totally sclerotic glomeruli	1.4±2.8	1.07±2.3	1.3±2.6	0.16±0.4	2.2±3.5	2.4±3.8	1.4±1.3	0.152
Activity (%)	38±34	30±23	32.2±25	22.5±14	64±28	64.8±28	61±32	0.0001
Chronicity (%)	10.7±16	8.3±10	9.7±10	3.5±3.9	17.9±21	18.2±20	16.5±26	0.036
Number of glomeruli with crescents	1.1±2	0.61±0.98	0.65±1	0.5±0.8	2.3±2.9	2.3±2.7	2.3±3.9	0.005
Proportion of endocapillary proliferation	0.67±0.4	0.76±0.4	0.8±0.4	0.6±0.51	0.94±0.2	0.9±0.2	1±0	0.03

*Comparison Class III and Class IV was made by two-sample t test.

## 5. Discussion

This study looked at the correlation of various demographic and laboratory findings in cases of biopsy-proven LN with the pathomorphological features on renal biopsies as observed under the LM. Lupus nephritis remains one of the most severe manifestations of SLE associated with substantial morbidity and mortality. Around 60% of adults and 80% of children with SLE develop LN (1-3,7-11). The demographic characteristics of our study cohort conform to those previously reported from around the world. The mean age of the patients is young as reported previously. The gender distribution is also biased in favor of females, as is well established in almost all previous studies on LN, and in a study from neighboring province in Iran (1-3,11). There was no significant difference in the degree of proteinuria between two classes of III and IV LN. In this study, a significant association of serum creatinine with the degree of proteinuria was seen. Serum creatinine also had significant positive correlation with the percent activity, percent chronicity and also with the proportion of glomeruli with crescents. While, there was a significant relationship of proteinuria with chronicity percent, this association for activity percent was not significant. Among all the lesions, the extracapillary proliferation (crescent formation) is a histopathologic marker of severe kidney damage, defined by the disruption in the integrity of the glomerular capillary wall, leading to macrophage and T cell inﬁltration into the Bowman’s space, with further aggravation of the inﬂammatory process and the glomerular damage (5,7,8). In our observational study, crescents were found in 42.86 % of patients. In our study, serum creatinine had significant positive correlation with the proportion of glomeruli with crescents, revealing the significance of extracapillary proliferation. Chen *et al.* studied 520 diffuse proliferative lupus nephritis patients (5). They found that the crescentic type of diffuse proliferative lupus nephritis had shorter LN duration, higher prevalence of rapidly progressive glomerulonephritis (RPGN) syndrome, and gross hematuria. This group also had more severe hypoproteinemia, hyperlipidemia, and renal failure, heavier proteinuria and microscopic hematuria and also higher tubular injury parameters. They also detected that, the proportion of death, end-stage kidney disease, and treatment failure correlates positively with the degree of histological crescents (5). They finally concluded that, patients with crescentic LN who presented with acute onset and short disease duration, mostly show intense kidney manifestations, serious capillary necrosis, severe tubulointerstitial inflammation, atrophy and fibrosis, prominent leukocyte infiltration, poor treatment response, and worse renal outcome (5). Likewise, Vandepapelière *et al*., conducted a study to evaluate whether the different ISN/RPS classes of proliferative lupus nephritis have a distinct baseline presentation (9). They studied ninety-eight patients with new onset ISN/RPS proliferative lupus nephritis. They found that the baseline serum creatinine and 24-hour proteinuria were higher in class IV-global, as was activity index on kidney biopsy in class IV-segmental and IV-global compared to class III (9). While, we detected, endocapillary proliferation in 66.67% of all patients, in a study on 71 biopsy-proven LN patients, Shariati-Sarabi *et al.* found endocapillary hypercellularity in 83.1% of their patients (10). They also found that, patients with kidney failure had been more frequently involved with fibrous crescents (10). Additionally, 82.14% of our patients were female, which was similar to previous study conducted by Nezhad *et al*., in the neighboring province of our country. They found that of 144 LN patients, 84.7 % were females (11).

## 6. Conclusions

In conclusion, the results from our study on biopsy-proven cases of LN largely concur with the previously reported studies from Iran and other parts of the world. The clinical and morphological significance of some lesions such as crescents in LN needs further investigation in future studies.

## Authors’ contributions

All authors wrote the paper equally.

## Conflict of interests

The authors declared no competing interests.

## Funding/Support

None.
